# Cytotoxic and Antimicrobial Compounds from the Marine-Derived Fungus, *Penicillium* Species

**DOI:** 10.3390/molecules23020394

**Published:** 2018-02-12

**Authors:** Diaa T. A. Youssef, Abdulrahman M. Alahdal

**Affiliations:** 1 Department of Natural Products, Faculty of Pharmacy, King Abdulaziz University, Jeddah 21589, Saudi Arabia; 2 Department of Clinical Pharmacy, Faculty of Pharmacy, King Abdulaziz University, Jeddah 21589, Saudi Arabia; aalahdal2@hotmail.com

**Keywords:** marine *Penicillium* sp., 2,5-diketopiperazines, cytotoxic and antimicrobial activities

## Abstract

The organic extract of liquid cultures of the marine-derived *Penicillium* sp. was investigated. Fractionation of the extracts of the fungus led to the purification and identification of two new compounds, penicillatides A (**1**) and B (**2**), together with the previously reported cyclo(*R*-Pro*–S*-Phe) (**3**) and cyclo(*R*-Pro*–R*-Phe) (**4**). The structures of compounds **1**–**4** were assigned by extensive interpretation of their NMR and high-resolution mass spectrometry (HRMS). The antiproliferative and cytotoxic activities of the compounds against three human cancer cell lines as well as their antimicrobial activity against several pathogens were evaluated. Compounds **2**–**4** displayed variable cytotoxic and antimicrobial activities.

## 1. Introduction

Members of the genus *Penicillium* are among the most investigated fungi by natural products chemists and are considered a major source for drug discovery. Prominent fungal-derived drugs include the antibiotic penicillin, which was obtained from *Penicillium chrysogenum*, and the antifungal griseofulvin from *Penicillium griseofulvum*. Nowadays, the genus *Penicillium* still represents a major producer of secondary metabolites with diverse bioactivities, as reported in many recent reviews [1,2,3,4,5].

In the last decade, a significantly increased interest in secondary metabolites from marine microbes has been reported [2]. 2,5-Diketopiperazines (2,5-DKPs) are obtained from different organisms, including marine microbes. They represent an important group of cyclic dipeptides with diverse structures and significant biological activities [2]. Since the number of reported compounds with significant biological properties is increasing, several reviews covering the structural determinations, biological activities, and proposed biosynthetic pathways of this class of compounds have been reported [6,7,8]. Consequently, marine fungi appear to be a promising source of these interesting dipeptides. Several members of the 2,5-DKP class displayed cytotoxic, anti-inflammatory, and antimicrobial activities [9,10,11,12].

**Figure 1:**
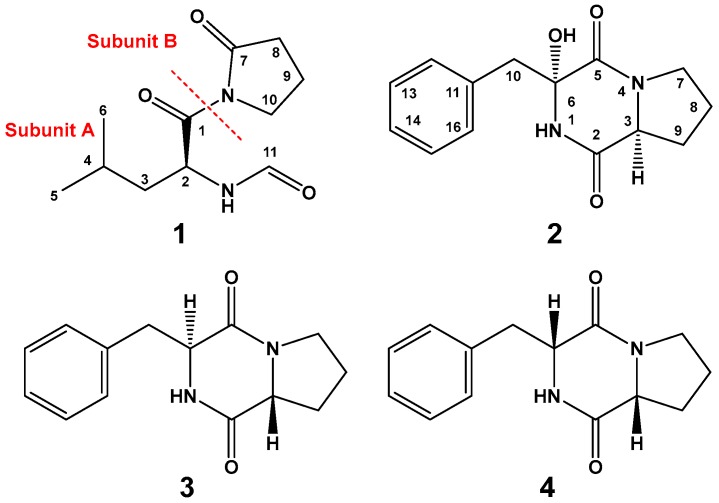
Chemical structures of compounds **1**–**4**.

Our growing interest in identifying secondary metabolites from marine microbes resulted in the identification of several compounds with different bioactivities [13,14,15,16,17]. The organic extract of the fungus *Penicillium* sp. gave two new compounds, named penicillatides A and B (**1** and **2**), together with cyclo(d-Pro–l-Phe) {cyclo(*R*-Pro–*S*-Phe)} (**3**) [18] and cyclo(*R*-Pro–*R*-Phe) (**4**) [19] (Figure 1). Structural determinations of the compounds were supported by interpretation of their spectroscopic data as well as comparison of available NMR data in the literature. In this paper, the structural determinations and the cytotoxic and antimicrobial activities of the compounds are reported.

## 2. Results and Discussion

### 2.1. Structure Elucidation of Compounds ***1**–**4***

**Figure 2:**
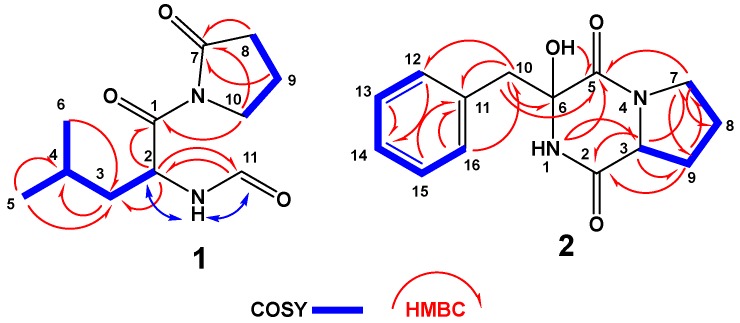
Key COSY and HMBC correlations of **1** and **2**.

The high-resolution electrospray ionization mass spectrometry (HRESIMS) and NMR spectra (Figures S1–S4) of compound **1** (Figure 1) support the molecular formula of C_11_H_18_N_2_O_3_. The ^13^C-NMR (Figure S2) spectrum of **1** (Figure S2) displayed signals for 11 carbons, including two methyls, four methylenes, two aliphatic methines, one aldehydic methine, and two quaternary amidic carbonyls (Table 1). Tracing of the ^1^H–^1^H couplings in the COSY spectrum (Figure S3) allowed the assignment of two structural subunits in **1**: *N*-formylleucine (subunit A) and 2-oxopyrrolidine (subunit B) moieties (Figure 1 and Table 1). The first ^1^H–^1^H COSY coupling system led to the assignment of the *N*-formyl-leucine moiety as established from the multiplicity-edited HSQC spectral data (Figure S4) at δ_H/C_ 6.10 (N*H*), 173.7 (qC, C-1), 5.81/49.9 (CH, H-2/C-2), 1.62, 1.42/41.5 (CH_2_, H_2_–3/C-3), 1.74/25.0 (CH, H-4/C-4), 1.05/21.0 (CH_3_, H_3_–5/C-5), 0.92/23.5 (CH_3_, H_3_-6/C-6), and 8.21/160.6 (CH, H-11/C-11) (Table 1). The ^1^H/^13^C chemical shift at δ_H/C_ 8.21/160.6 is typical for an *N*-formyl moiety and in agreement with reported values [20]. The placement of the *N*-formyl moiety was supported by HMBC of H-2/C-11, H-11/C-2 as well as NOESY correlations of N*H*/H-2 and N*H*/H-11 (Figure 2). Further HMBC correlations (Figure S5) within the leucine moiety are shown in Table 1 and Figure 2. The second coupling system allowed the assignment of the 2-oxopyrrolidine subunit based on the continuous ^1^H–^1^H COSY coupling system of H_2_-8/H_2_-9/H_2_-10 and the ^1^H/^13^C NMR signals at δ_H/C_ 174.8 (qC, C-7), 2.64, 2.61/33.4 (CH_2_, H_2_-8/C-8), 2.09/17.3 (CH_2_, H_2_-9/C-9), and 3.88, 3.77/45.6 (CH_2_, H_2_-10/C-10). The HMBC correlations of H_2_-8/C-7, H_2_-9/C-7, and H_2_-10/C-7 supported the assignment of subunit B (Figure 2). Finally, the connection between the two subunits of **1** at C-1 and the amidic nitrogen of the 2-oxopyrrolidine moiety was secured from the HMBC correlation from H_2_-10 to C-1 (Figure 2), supporting the assignment of **1** as *N*-(*N*-formylleucyl)-2-oxopyrrolidine.

**Scheme 1:**
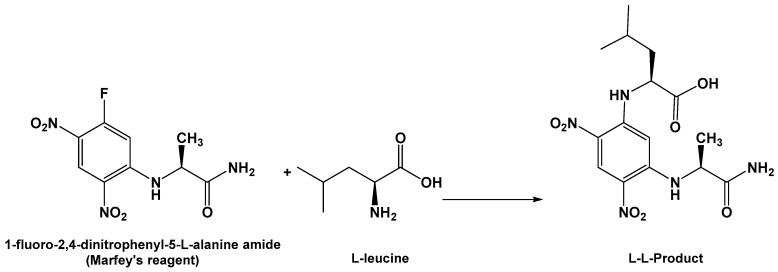
Reaction of 1-fluoro-2,4-dinitrophenyl-5-l-alanine amide (FDAA) with the hydrolytic product of compound **1** (l-leucine).

The absolute configuration of C-2 of the leucine residue was determined by Marfey’s method (Scheme 1) [21]. HPLC analysis of the derivative of 1-fluoro-2,4-dinitrophenyl-5-l-alanine amide (FDAA, Marfey’s reagent) with the hydrolytic product of **1** gave the same retention time as the derivative prepared from an authentic l-leucine, and therefore the l-configuration was assigned to the leucine residue. Thus, compound **1** was assigned as (S)-*N*-(4-methyl-1-oxo-1-(2-oxopyrrolidin-1-yl)pentan-2-yl)formamide. The name penicillatide A was given to **1**, and it is reported here as a new natural compound.

**Scheme 2:**
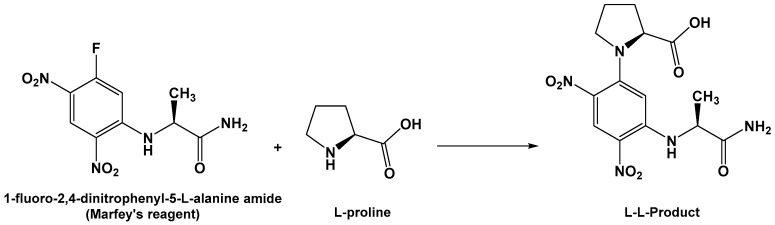
Reaction of FDAA with the hydrolytic product of compound **2** (l-proline).

**Figure 3:**
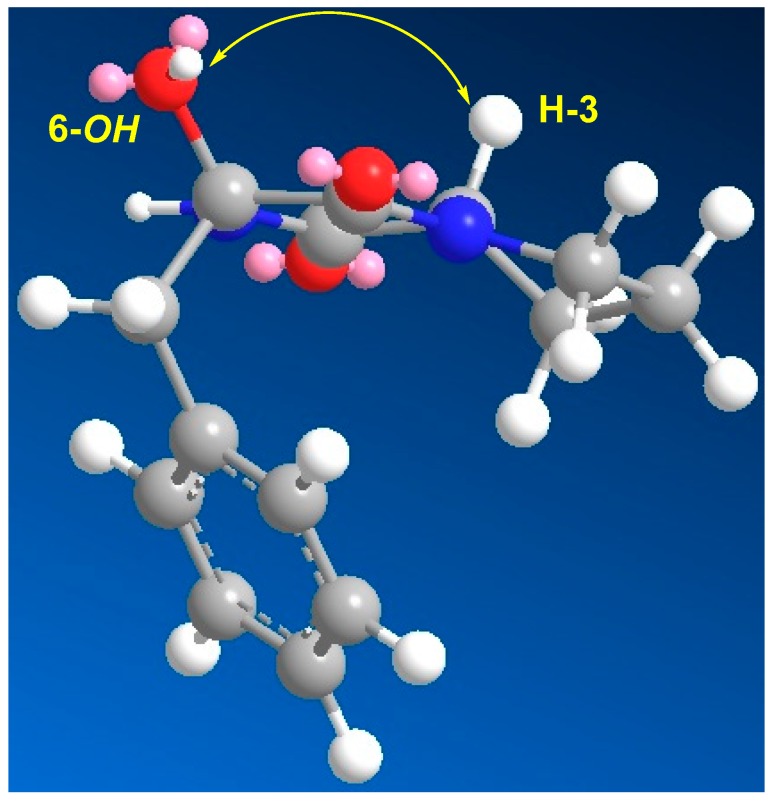
MM2 energy-minimized drawing of **2** showing an important NOE correlation.

Compound **2** (Figure 1) exhibited a molecular formula of C_14_H_16_N_2_O_3_ as established by HRESIMS. The ^1^H/^13^C NMR signals (Figures S6 and S7, Table 2) at δ_H/C_ 6.13 (1H, brs, N*H*-1), 166.9 (qC, C-2), 2.85/59.6 (CH, H-3/C-3), and 166.1 (qC, C-5) suggested a diketopiperazine moiety as part of a cyclic dipeptide skeleton. The NMR data of compound **2** (Table 2) are very close to those of 3. However, compound **2** displayed a signal for an oxygenated quaternary carbon at δ_C_ 83.4 and ^1^H singlet at δ_H_ 4.36 (1H, O*H*) instead of the methine of H-6/C-6 at δ_H/C_ 4.20/59.1 in **3**, suggesting the presence of a hydroxyl moiety at C-6 in **2**. The placement of the OH at C-6 was supported by HMBC correlations of H_2_-10/C-6, H_2_-10/C-5, and O*H*/C-5 (Figure 2 and Table 2). Furthermore, the COSY (Figure S8), HSQC (Figure S9) and HMBC experiments (Figure S10) supported the assignment of the structure of **2** as cyclo(Pro–2-O*H–*Phe) (Table 2 and Figure 2). The absolute configuration of C-3 of the proline residue was determined by Marfey’s method [21]. HPLC analysis of the FDAA derivative of the hydrolytic product of **2** gave the same retention time as the derivative prepared from an authentic (l)-proline, and therefore the l-configuration was assigned to the proline residue (Scheme 2). The absolute configuration at C-6 of the phenylalanine residue was not determined by Marfey’s method, because the hydroxyphenylalanine residue decomposed under acidic conditions. The relative configuration at C-6 was established by comparing the chemical shift values of C-6 and H_2_-10/C-10 with a similar compound containing the *α*-hydroxylated phenylalanine moiety, namely (3*R*,6*S*)-3-benzyl-3-hydroxy-1,4-dimethyl-6-((4-nitro-1*H*-indol-3-yl)methyl)piperazine-2,5-dione [22]. The δ_H/C_ of 3.14, 3.05/47.8 (H_2_-10/C-10), and 83.4 (C-6) in **2** were very close to the values in the literature [22], supporting the configuration of OH at C-6, as shown in Figure 1. Furthermore, the configuration of the OH at C-6 in **2** was also confirmed by the NOESY experiment, in which a strong correlation between H-3 (δ_H_ 2.85) and 6-OH (δ_H_ 4.36) was observed (Figure 3). Further studies are needed to determine the absolute configuration at C-6 in **2**. Thus, the relative configuration at C-6 was assigned as R*. Accordingly, compound **2** was assigned as (3R*,8*aS*)-3-benzyl-3-hydroxyhexahydropyrrolo[1,2-*a*]pyrazine-1,4-dione. The name penicillatide B was given to compound **2**, and it is reported here as a new natural product.

Compounds **3** and **4** were assigned as cyclo(d-Pro–l-Phe) {cyclo(*R*-Pro–*S*-Phe)} (**3**) [18] and cyclo(*R*-Pro–*R*-Phe) (**4**) by interpretation of their NMR (Figures S11–S20) and HRMS data.

### 2.2. Biological Activities of Compounds ***1**–**4***

Compounds **2**–**4** were evaluated for their cytotoxic and antiproliferative activities against colorectal carcinoma (HCT 116), hepatocellular carcinoma (HepG2), and breast cancer (MCF-7) using a sulforhodamine assay, as previously described [23]. Compounds **2** and **3** showed significant activity against HCT-116, with IC_50_ of 23.0 and 38.9 µM, while compound **4** was weakly active against this cell line. Compounds **3** and **4** were weakly active against MCF-7, with IC_50_ of 102 and 104 µM, respectively. Finally, none of the compounds showed activity against HepG2 (≥50 µM).

In addition, **2**–**4** were evaluated for their antimicrobial activity against several microbes: *S. aureus*, *Vibrio anguillarum*, and *C. albicans*. Compounds **2**–**4** showed significant activity against *V. anguillarum*, with inhibition zones of 20, 24, and 25 mm, respectively. Similarly, compounds **2**–**4** showed moderate activity against both *S. aureus* and *C. albicans*, with inhibition zones between 10 and 19 mm (Table 3).

The results in Table 3 clearly show that compounds **2**–**4** were selective against HCT-116 without any activity against HepG2. In comparison to **3** and **4**, the hydroxylation of C-6 (in **2**) potentiated the cytotoxic activity against HCT-116. In the antimicrobial screen, **2**–**4** were almost identically active against *S. aureus* and *C. albicans,* suggesting no effect for the OH at C-6. In addition, **2** and **3** were more active than **2** against *V. anguillarum* and showed similar activity to the positive control ciprofloxacin.

## 3. Materials and Methods

### 3.1. General Experimental Procedures

One- and two-dimensional NMR spectra (chemical shifts in ppm, coupling constants in Hz) were recorded on Bruker Avance DRX 600 MHz (Bruker, Rheinstetten, Germany) and Bruker Ascend™ 850 (850 MHz) (Bruker BioSpin, Billerica, MA, USA) spectrometers using CDCl_3_ as solvent. NMR spectra were referenced to the residual protonated solvent signals (CHCl_3_: 7.26 ppm for ^1^H and 77.0 ppm). Positive ion HRESIMS data were obtained with a Micromass Q-ToF equipped with leucine enkephalin lockspray, using *m*/*z* 556.2771 (M + H)^+^ as a reference mass. For column chromatography, silica gel (Merck, 70–230 mesh ASTM, Sigma-Aldrich, Darmstadt, Germany) and Sephadex LH-20 (0.25–0.1 mm, Pharmacia, Piscataway, NJ, USA) were used. Precoated silica gel 60 F-254 plates (Merck) were used for TLC. HPLC purifications were performed on a semipreparative HPLC column (RP18, 5 μm, ARII Cosmosil, 250 × 10 mm, Waters, Nacalai, Inc., San Diego, CA, USA).

### 3.2. Biological Materials

The marine-derived fungus *Penicillium* sp. was isolated from the Red Sea tunicate *Didemnum* sp., and the fungus was identified as previously described [13].

### 3.3. Culture Condition and Extraction

Large-scale culture of the fungus *Penicillium* sp. was carried out in 20 flasks (each 2 L). Each flask contained 500 mL of Sabouraud Dextrose (HiMedia Laboratories, Vadhani Ind. Est., LBS Marg, Mumbai, India) Broth (SDB) liquid medium. The prepared liquid cultures were shaken on an orbital shaker at 28 °C continuously for 14 days. After 2 weeks of shaking and incubation, the cultures were filtered using clean gauze to separate the formed fungal mycelia from the broth. The culture broth from each flask was extracted with EtOAc (3 × 300 mL). The mycelia formed during the shaking were lyophilized and extracted with MeOH. The ethyl acetate and methanolic extracts were combined and evaporated under vacuum, and the resulting extracts were used for fractionation and purification of the compounds.

### 3.4. Isolation and Purification of Compounds ***1**–**4***

The combined extracts of the broth and mycelia (0.76 g) were flash chromatographed on reverse-phase SiO_2_ using H_2_O-MeOH gradients, giving 6 subfractions (A–F). Fraction B (120 mg), eluted with 25% MeOH in H_2_O, was fractionated on Sephadex LH-20 column using MeOH as eluent, giving 6 main subfractions (B1–B6). Fraction B3 (56 mg) was purified on C18 HPLC column using 50% CH_3_CN to give compounds **3** (5.5 mg) and **4** (3.5 mg). Similarly, subfraction B4 (30 mg) was purified on C18 HPLC column using 45% CH_3_CN to give compounds **1** (2.7 mg) and **2** (2.3 mg).

### 3.5. Determination of Configuration of the Amino Acids in ***1*** and ***2***

About 0.5 mg each of compounds **1** and **2** was heated separately in 1 mL of 6 N HCl at 100 °C for 16 h, followed by removal of the excess HCl under vacuum. To each dry hydrolysate, 200 µL of 1% solution of FDAA [21] in acetone and 40 µL of 1.0 M NaHCO_3_ were added. The reaction mixture was heated at 45 °C for 1.5 h, cooled, and acidified with 20 µL of 2.0 M HCl. Similarly, standard amino acids (d and l) of leucine and proline were derivatized separately. The derivatized standard amino acids and hydrolysates of **1** and **2** were subjected to HPLC on Nova-Pak C18 reverse-phase column (150 × 3.9 mm i.d., 4 mm particle size; Waters, Milford, MA, USA) using the following gradient program. Solvent A was a 50 mM triethylamine–phosphate buffer (pH 3.5) containing 25% (*v*/*v*) MeOH, and solvent B was the same buffer containing 70% MeOH. The mobile phase was a linear gradient from 0 to 100% B (100 to 0% A) in 40 min, at a flow rate of 0.65 mL/min at 25 °C. The eluted peaks were monitored at 340 nm. The retention times for FDAA derivatives of standards and compounds **1** and **2** were as follows: (l)-leucine (*t*_R_ 27.2 min), (d)-leucine (*t*_R_ 36.0 min), (l)-proline (*t*_R_ 15.4 min), (d)-proline (*t*_R_ 19.1 min), compound **1** (*t*_R_ 27.2 min), and compound **2** (*t*_R_ 15.4 min).

*Penicillatide A* (**1**): White amorphous powder; (α)_D_ = −22° (*c* 0.05, MeOH); NMR data (Table 1); HRESIMS *m*/*z* 227.1395 (calcd for C_11_H_19_N_2_O_3_, (M + H)^+^, 227.1396).

*Penicillatide B* (**2**): White amorphous powder; (α)_D_ = 85° (*c* 0.08, MeOH); NMR data (Table 2); HRESIMS *m*/*z* 261.1240 (calcd for C_14_H_17_N_2_O_3_, (M + H)^+^, 261.1239).

### 3.6. Biological Activities of the Compounds

#### 3.6.1. Evaluation of the Cytotoxic Activities of the Compounds

The cytotoxicity of compounds **2**–**4** against 3 tumorous cell lines—colorectal carcinoma, breast cancer, and hepatocellular carcinoma—were evaluated using sulforhodamine assay [23]. Briefly, before adding the compounds, the cells were grown in 96-well plates for 24 h. After adding the compounds, incubation of the cells was carried out for another 48 h. The IC_50_ values of the compounds were obtained from the log dose–response curve. The reported IC_50_ values were obtained from the means of 3 experiments.

#### 3.6.2. Antibacterial Evaluation of the Compounds

A disc diffusion assay was used to determine the antimicrobial activity of the compounds [24] with replication (*n* = 3). *Staphylococcus aureus*, *Vibrio anguillarum*, and *Candida albicans* served as target models for bacteria and fungi. A total of 100 μg of each compound was loaded onto 6-mm sterile circular filter-paper discs. The paper discs were left to air-dry. The dried paper discs were placed onto nutrient agar plates that had already been inoculated with a lawn of target microorganisms. After 24 h of incubation, the antimicrobial activity of the compounds was calculated.

## 4. Conclusions

Investigation of the marine-derived fungus *Penicillium* sp. gave two new compounds, penicillatides A and B (**1** and **2**), together with the known compounds cyclo(*R*-Pro–*S*-Phe) (**3**) and cyclo(*R*-Pro–*R*-Phe) (**4**). The structures of **1**–**4** were assigned by interpretation of their spectroscopic data by NMR and high-resolution mass spectroscopy (HRMS). Compounds **2** and **3** displayed significant and selective activity against HCT-116, with IC_50_ of 6.0 and 9.57 µg/mL, respectively, while they were inactive against HepG2 and MCF-7. These results suggest a selective effect of **2** and **3** against HCT-116. Also, **2**–**4** showed potent antimicrobial activity against *V. anguillarum*, with inhibition zones of 20, 24, and 25 mm, respectively. On the other hand, **2**–**4** were moderately active against *S. aureus* and *C. albicans*.

## Figures and Tables

**Table 1:** NMR data of compound **1** (CDCl_3_, 850 and 213 MHz).

No.	δ_C_ (mult.)	δ_H_ (mult., *J* (Hz))	HMBC	NOESY
**1**	173.7, qC		H-2, H_2_-10	
**2**	49.9, CH	5.81 (dt, 11.5, 3.0)	H-11	N*H*
**3**	41.5, CH_2_	1.62 (m), 1.42 (m)	H-2, H_3_-5, H_3_-6	
**4**	25.0, CH	1.74 (m)	H_3_-5, H_3_-6	
**5**	21.0, CH_3_	1.05 (d, 6.5)	H_3_-6	
**6**	23.5, CH_3_	0.92 (d, 6.5)	H_3_-5	
**7**	174.8, qC		H_2_-8, H_2_-9, H_2_-10	
**8**	33.4, CH_2_	2.64 (dd, 9.5, 1.5) 2.61 (dd, 9.5, 7.0)	H_2_-9, H_2_-10	
**9**	17.3, CH_2_	2.09 (m)	H_2_-7, H_2_-8	
**10**	45.6, CH_2_	3.83 (m), 3.77 (m)	H_2_-8, H_2_-9	
**11**	160.6, CH	8.21 (s)	H-2	N*H*
**N*H***		6.10 (brs)		H-2, H-11

**Table 2:** NMR data of compound **2** (CDCl_3_, 600 and 150 MHz).

No.	δ_C_ (mult.)	δ_H_ (mult., *J* (Hz))	HMBC
**1 (N*H*)**		6.13 (brs)	
**2**	166.9, qC		H-1, H-3, H_2_-9
**3**	59.6, CH	2.85 (dd, 10.2, 6.8)	H-1, H_2_-8, H_2_-9
**5**	166.1, qC		H-1, H_2_-7, H_2_-10
**6**	83.4, qC		H_2_-10
**7**	45.5, CH_2_	3.63 (m) 3.41 (ddd, 12.8, 8.5, 2.5)	H-3, H_2_-9
**8**	22.1, CH_2_	1.98 (m), 1.71 (m)	H_2_-7
**9**	28.7, CH_2_	2.17 (m), 1.83 (m)	H-3, H_2_-8
**10**	47.8, CH_2_	3.14 (d, 13.6) 3.05 (d, 13.6)	H-12/H-16
**11**	133.1, qC		H_2_-10, H-12/H-16, H-13/H-15
**12**	130.6, CH	7.29 (m)	H-14
**13**	129.4, CH	7.40 (m)	H-14
**14**	128.1, CH	7.30 (m)	H-12/H-16, H-13/H-15
**15**	129.4, CH	7.40 (m)	H-14
**16**	130.6, CH	7.29 (m)	H-14
**O*H***		4.36 (brs)	

**Table 3:** Cytotoxic and antimicrobial activities of compounds **2**–**4**.

Compound	IC_50_ (µM)			Inhibition Zone (mm)@100 µg/disc		
	HCT-116	HepG2	MCF-7	*S. aureus*	*V. anguillarum*	*C. albicans*
**2**	23.0	≥50	≥50	19	20	10
**3**	38.9	≥50	102.0	14	24	11
**4**	94.0	≥50	114.0	16	25	15
**Doxorubicin ^a^**	0.789	0.621	0.415			
**Ciprofloxacin ^b^**				22	26	
**Ketoconazole ^c^**						30

^a^ Positive cytotoxic control; ^b^ positive antibacterial control (5 μg/disc); ^c^ positive antifungal control (50 μg/disc).

## Supplementary Materials

Supplementary materials are available online. Figures S1–S20 (1D and 2D NMR spectra of compounds **1**–**4**).
