# Supplementary material for: Cytotoxic and Antimicrobial Compounds from the Marine-Derived Fungus, Penicillium Species
Source: Molecules. 2018 Feb 12;23(2):394. doi: 10.3390/molecules23020394 (PMC6017105; doi:10.3390/molecules23020394)
Supplement: Supplementary file 1 [file molecules-23-00394-s001.pdf]

Supplementary information

Cytotoxic and Antimicrobial Compounds from the Marine-Derived Fungus, *Penicillium* Species

Diaa T.A. Youssef [1,*], and Abdulrahman M. Alahdal [2]

1 Department of Natural Products, Faculty of Pharmacy, King Abdulaziz University, Jeddah 21589, Saudi Arabia; dyoussef@kau.edu.
2 Department of Clinical Pharmacy, Faculty of Pharmacy, King Abdulaziz University, Jeddah 21589, Saudi Arabia; aalahdal2@hotmail.com
* Correspondence: dyoussef@kau.edu.sa; Tel.: +966-548-535-344

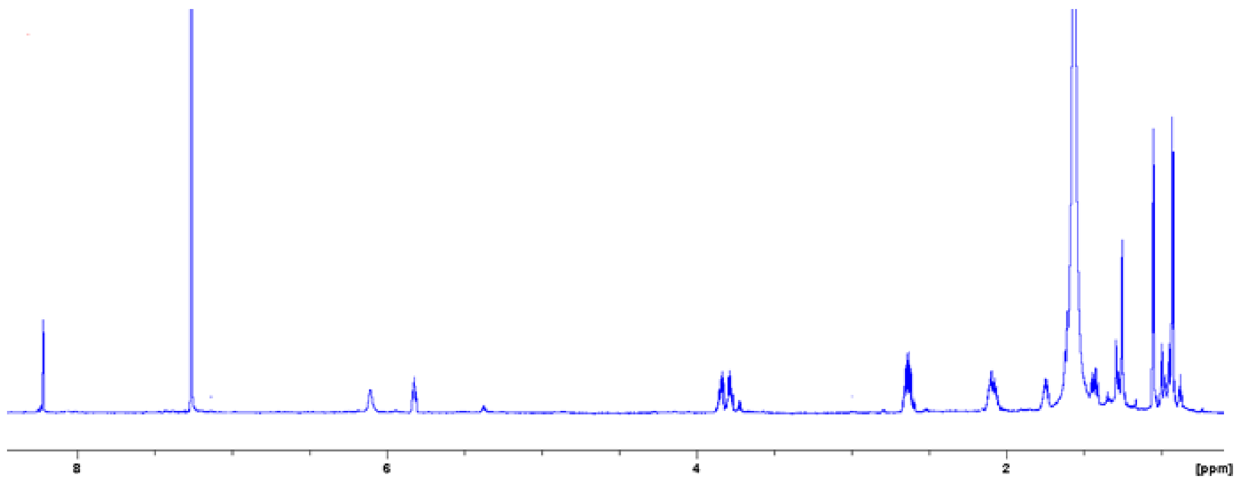

Figure S1. ^{1}H nuclear magnetic resonance (NMR) spectrum of compound **1** (850 MHz, $CDCl_3$).

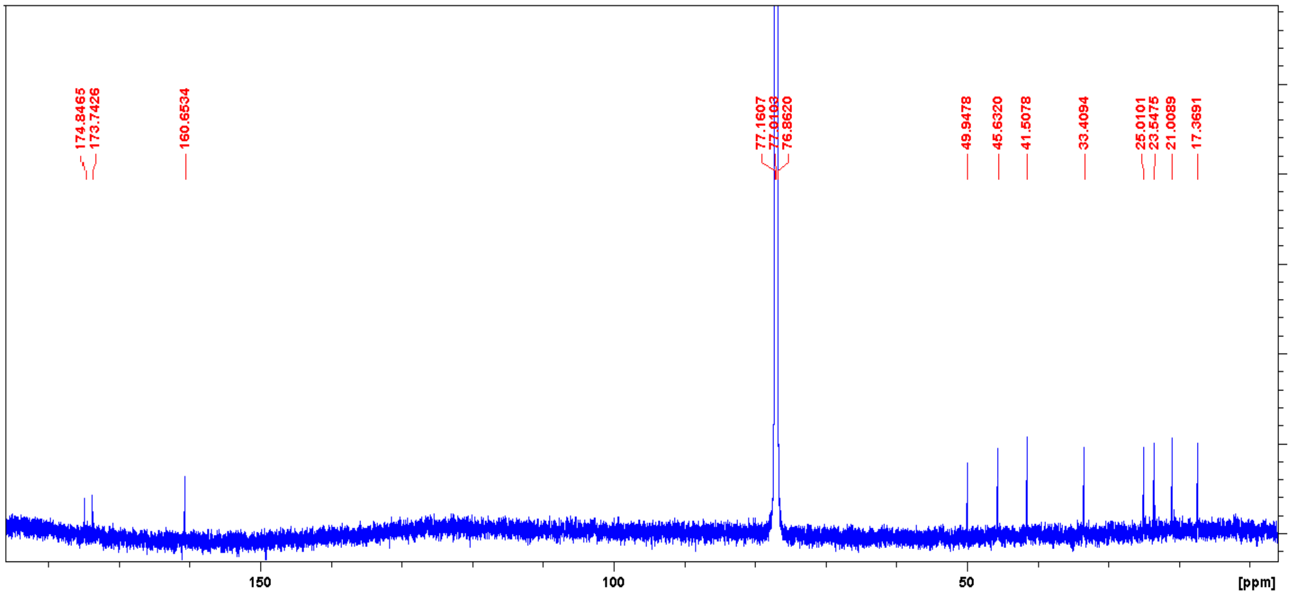

Figure S2. ^{13}C NMR spectrum of compound **1** (213 MHz, $CDCl_3$).

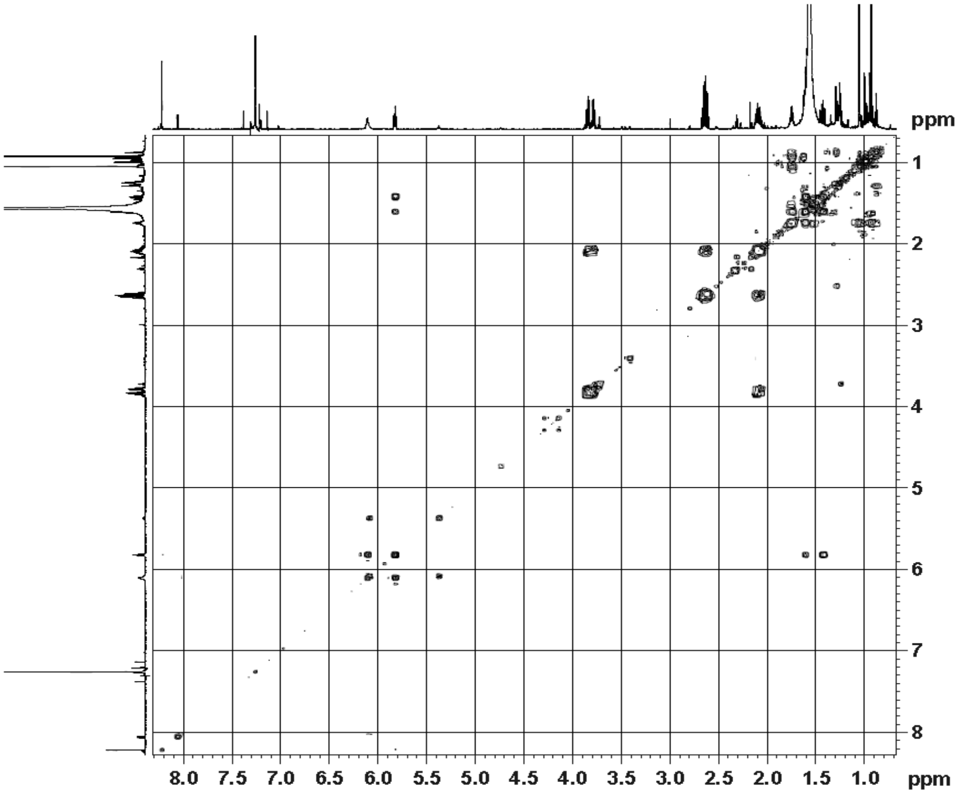

Figure S3. ^{1}H-^{1}H correlated spectroscopy (COSY) NMR spectrum of compound **1** (850 MHz, $CDCl_3$).

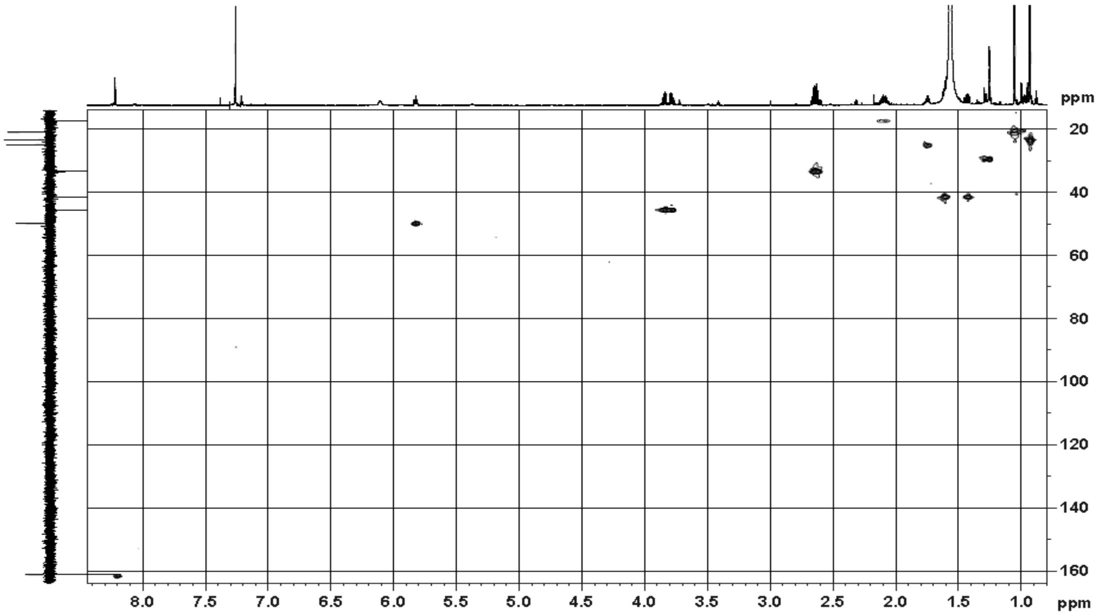

Figure S4. Multiplicity-edited heteronuclear single quantum coherence (HSQC) spectrum of compound **1** (850 MHz, $CDCl_3$).

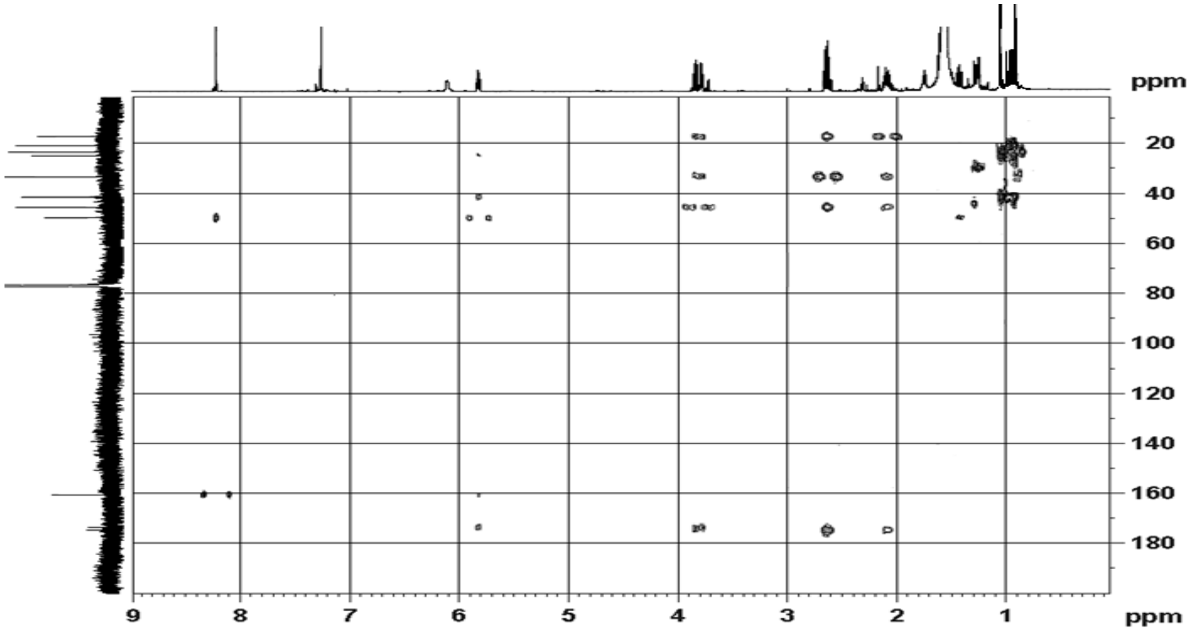

Figure S5. Heteronuclear multiple bond coherence (HMBC) spectrum of compound **1** (850 MHz, $CDCl_3$).

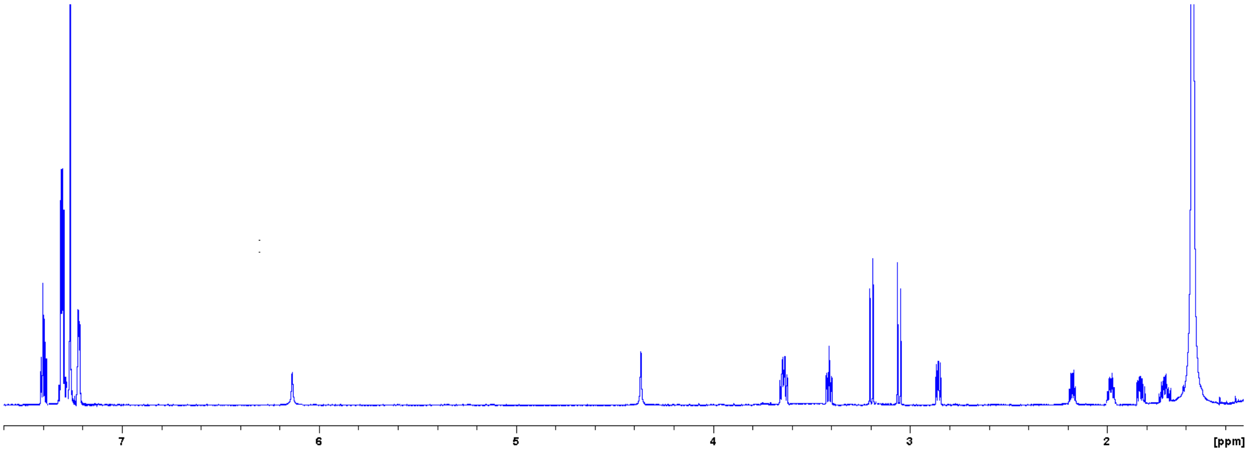

Figure S6. ^{1}H NMR spectrum of compound **2** (850 MHz, $CDCl_3$).

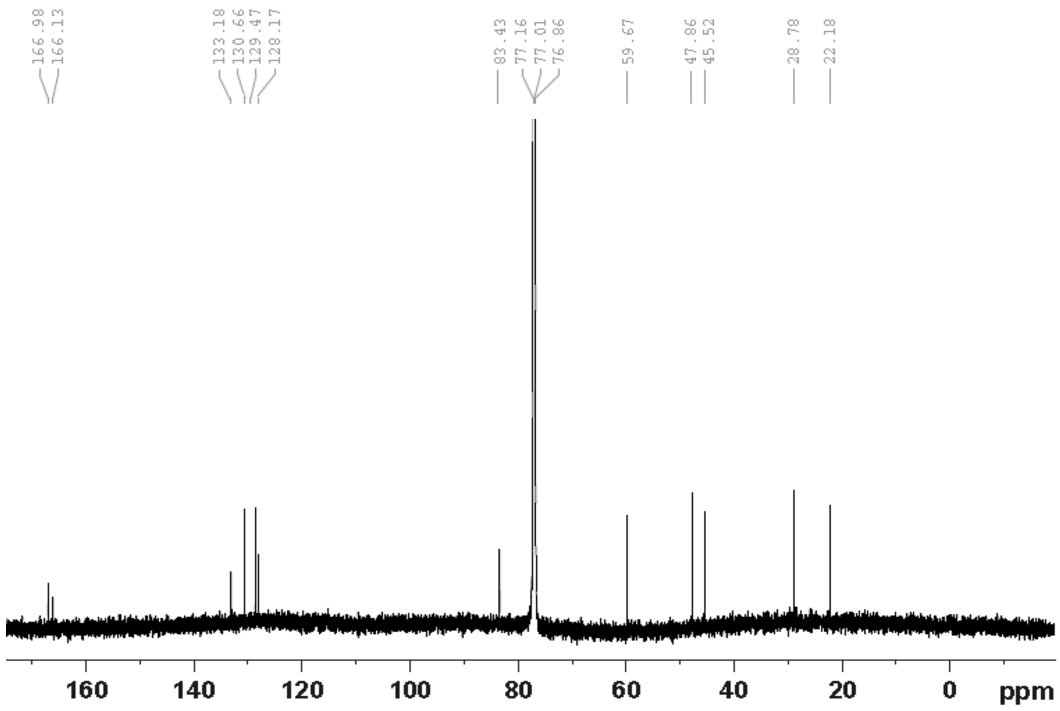

Figure S7. ^{13}C NMR spectrum of compound **2** (213 MHz, $CDCl_3$).

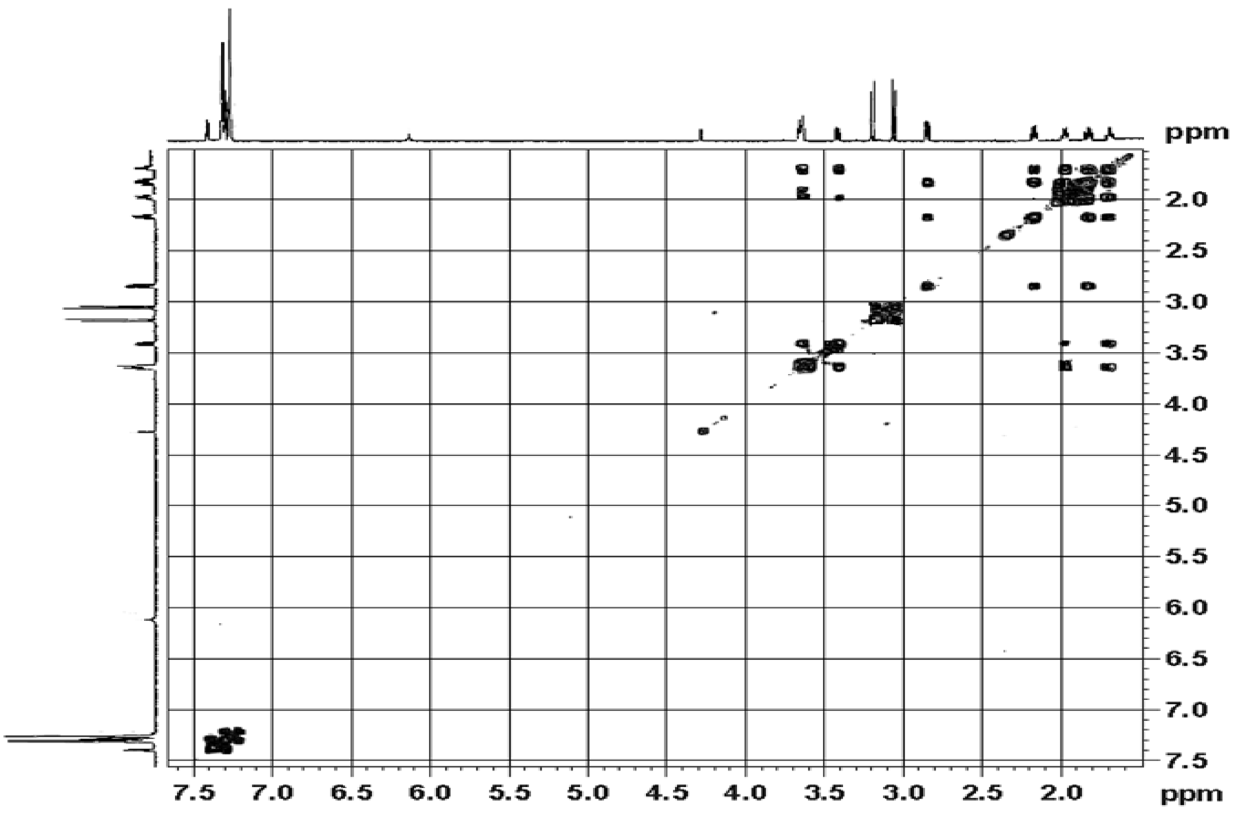

Figure S8. ^{1}H-^{1}H COSY spectrum of compound **2** (850 MHz, $CDCl_3$).

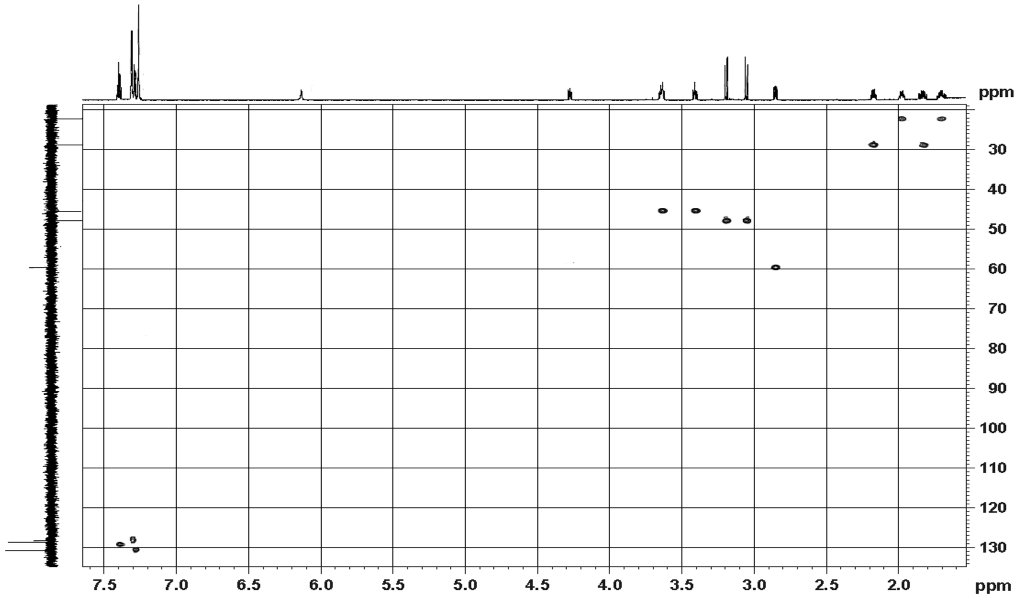

Figure S9. Multiplicity-edited HSQC spectrum of compound **2** (850 MHz, $CDCl_3$).

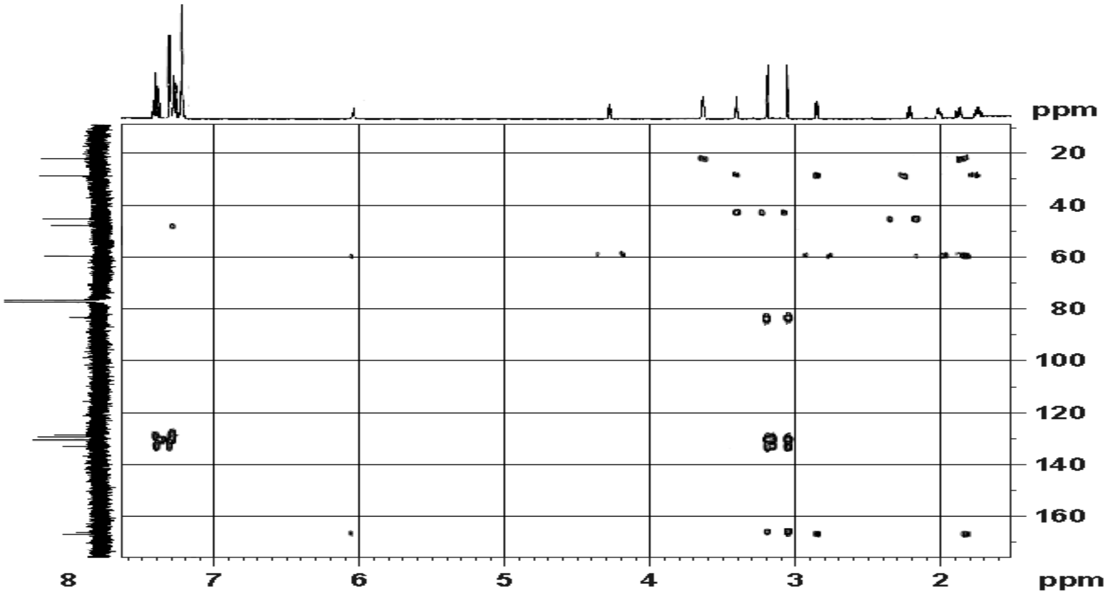

Figure S10. HMBC spectrum of compound **2** (850 MHz, $CDCl_3$).

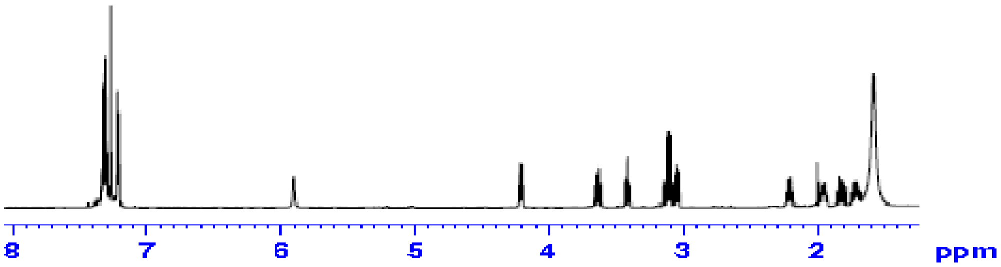

Figure S11. ^{1}H NMR spectrum of compound **3** (600 MHz, $CDCl_3$).

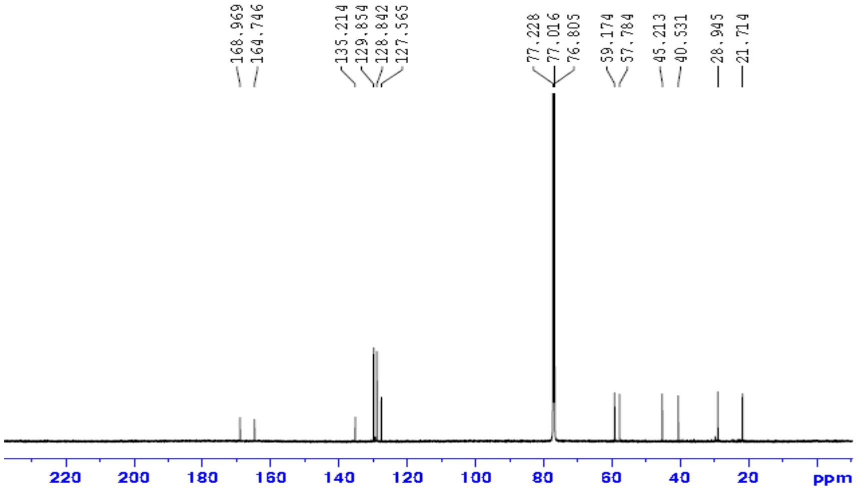

Figure S12. ^{13}C NMR spectrum of compound **3** (150 MHz, $CDCl_3$).

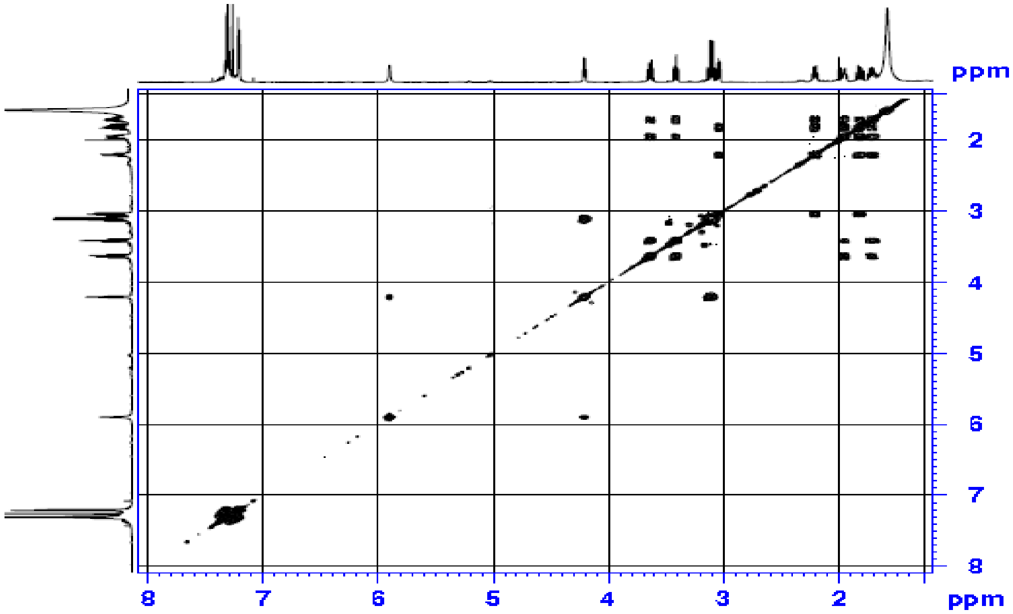

Figure S13. ^{1}H-^{1}H COSY spectrum of compound **3** (600 MHz, $CDCl_3$).

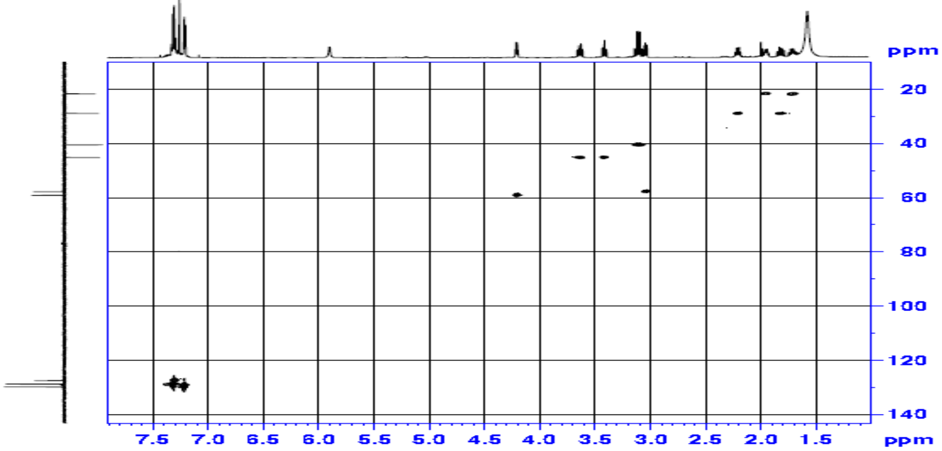

Figure S14. Multiplicity-edited HSQC spectrum of compound **3** (600 MHz, $CDCl_3$).

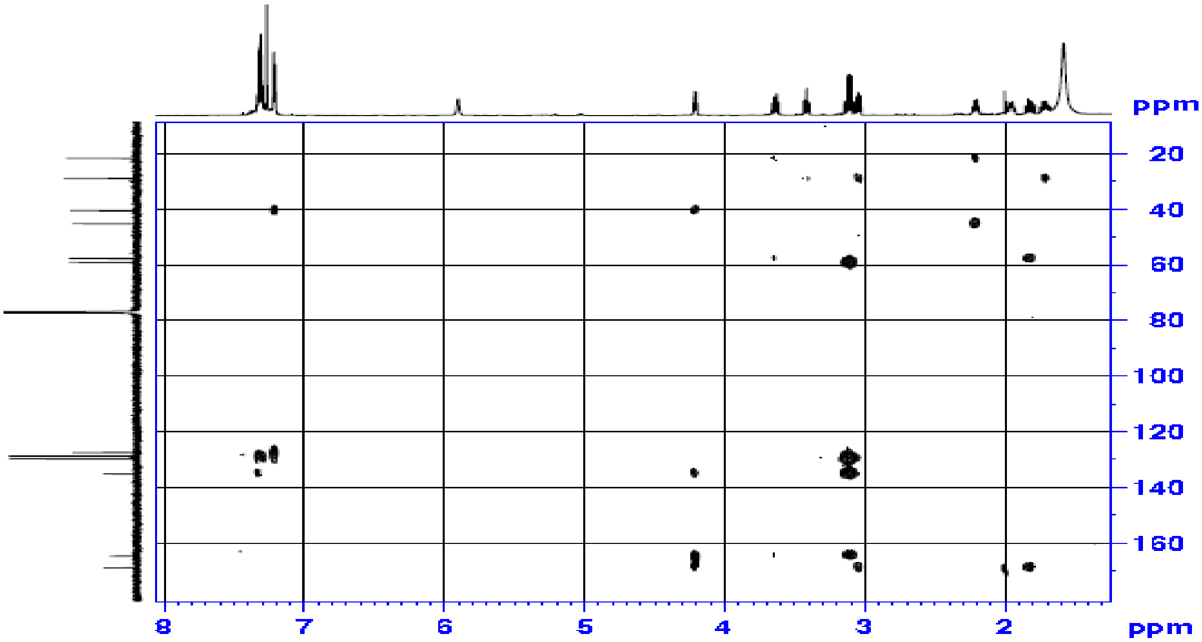

Figure S15. HMBC spectrum of compound **3** (600 MHz, $CDCl_3$).

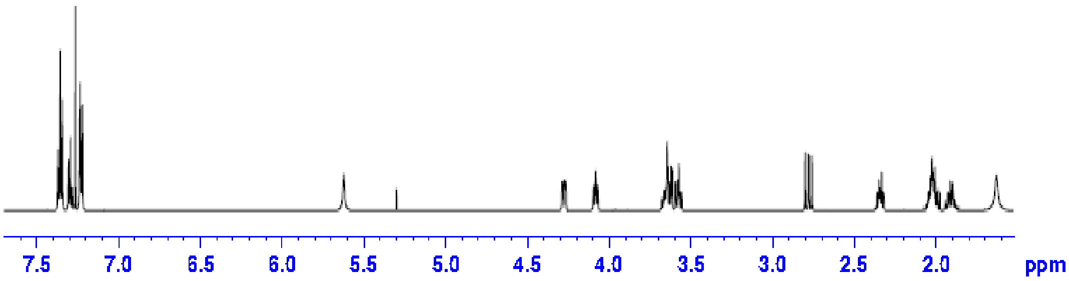

Figure S16. ^{1}H NMR spectrum of compound **4** (600 MHz, $CDCl_3$).

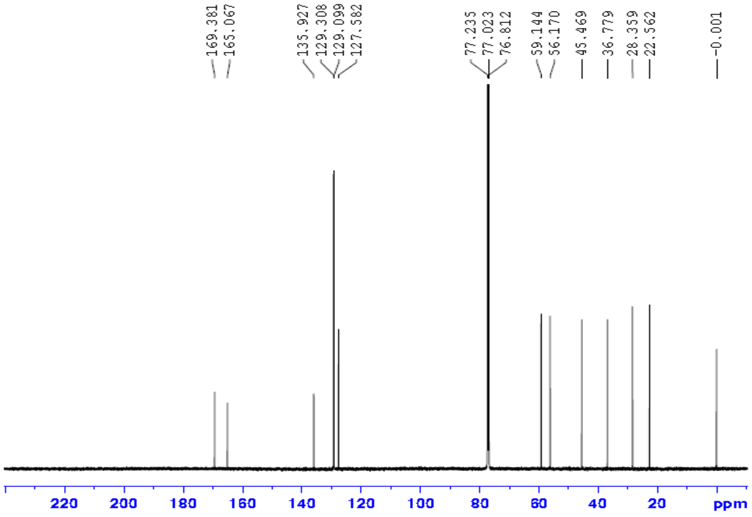

Figure S17. ^{13}C NMR spectrum of compound **4** (150 MHz, $CDCl_3$).

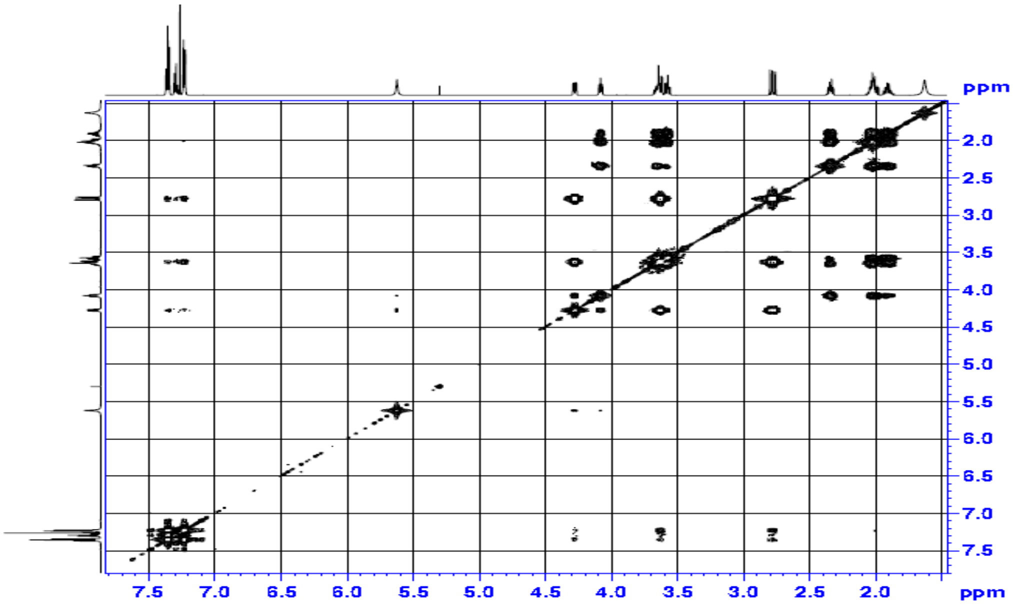

Figure S18. ^{1}H-^{1}H COSY spectrum of compound **4** (600 MHz, $CDCl_3$).

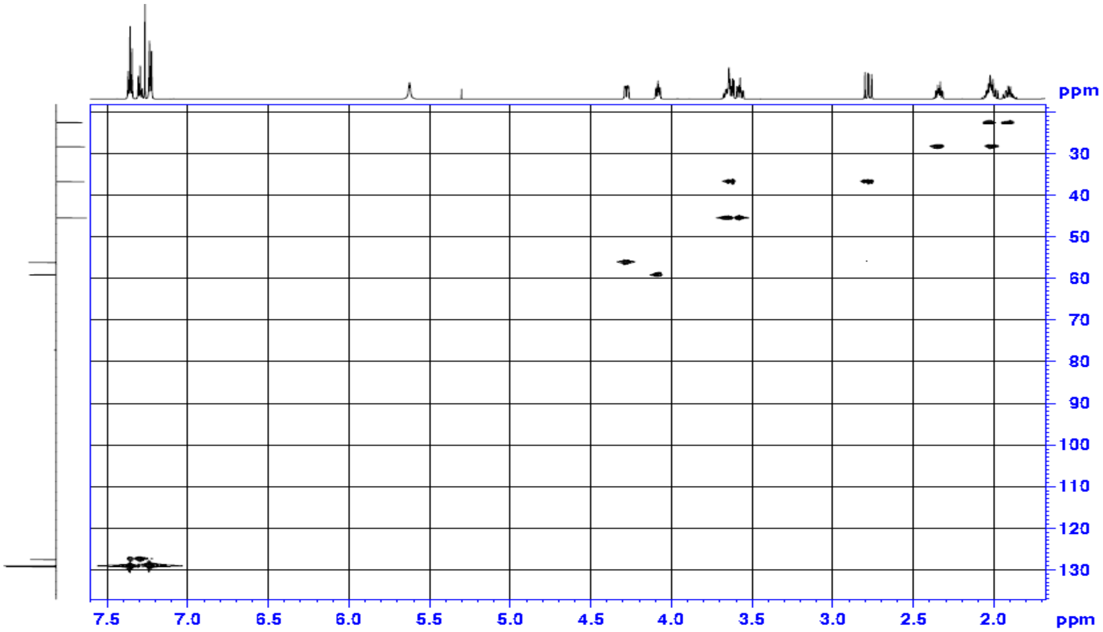

Figure S19. Multiplicity-edited HSQC spectrum of compound **4** (600 MHz, $CDCl_3$).

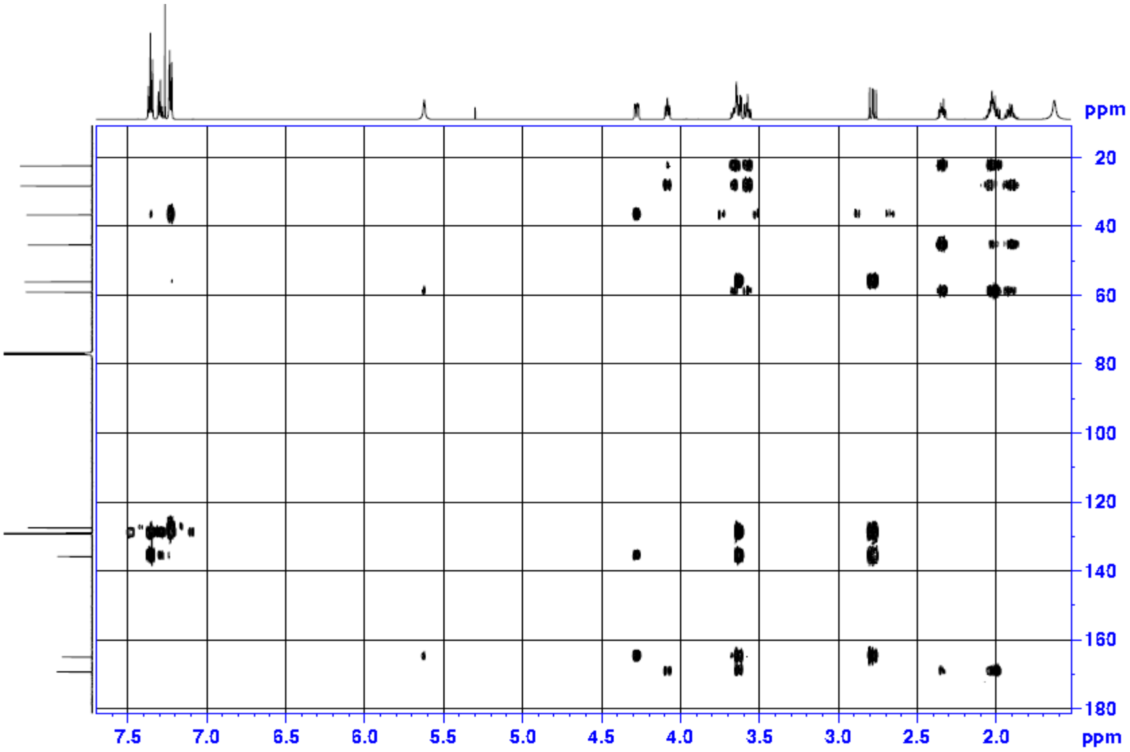

Figure S20. HMBC spectrum of compound **4** (600 MHz, $CDCl_3$).
